# Choose and Use Your Chemical Probe Wisely to Explore Cancer Biology

**DOI:** 10.1016/j.ccell.2017.06.005

**Published:** 2017-07-10

**Authors:** Julian Blagg, Paul Workman

**Affiliations:** 1 Cancer Research UK Cancer Therapeutics Unit, The Institute of Cancer Research, London SM2 5NG, UK

**Keywords:** chemical probe, chemical tool, target validation, pharmacological audit trail, biochemical selectivity, ligand promiscuity, Pan-Assay Interference Compounds, PAINS, chemical reactivity, lipophilicity, pharmacophore crossing

## Abstract

Small-molecule chemical probes or tools have become progressively more important in recent years as valuable reagents to investigate fundamental biological mechanisms and processes causing disease, including cancer. Chemical probes have also achieved greater prominence alongside complementary biological reagents for target validation in drug discovery. However, there is evidence of widespread continuing misuse and promulgation of poor-quality and insufficiently selective chemical probes, perpetuating a worrisome and misleading pollution of the scientific literature. We discuss current challenges with the selection and use of chemical probes, and suggest how biologists can and should be more discriminating in the probes they employ.

## Main Text

### The Value and Challenges of Chemical Probes

Chemical probes (or tools) are appropriately characterized small molecules, ideally of well-defined biological potency, selectivity, and cell permeability, which can be applied with confidence to interrogate complex biological systems. Numerous breakthroughs in biology have been enabled by the use of such small-molecule probes of sufficient quality, especially in concert with complementary biological reagents and molecular technologies. For example, the recent revolutionary growth in our understanding of bromodomain biology and pharmacology was triggered by the discovery of potent chemical probes JQ1 (Filippakopoulos et al., 2010), I-BET (Nicodeme et al., 2010), and their closely matched inactive partners used as controls, as well as subsequent companion probes targeting other bromodomain family members (Filippakopoulos and Knapp, 2014). Previously, the use of small-molecule chemical probes has helped drive increased fundamental understanding and therapeutic benefit across many biological areas, such as the cell cytoskeleton (colchicine and paclitaxel); mitotic spindle (monastrol); immunophilins and immunosuppression (FK506 and cyclosporin); mTOR signaling (rapamycin); histone/protein deacetylation (vorinostat); proteasome function (MG132); molecular chaperones (geldanamycin and radicicol); many protein kinases (from staurosporin and tyrphostins to numerous approved drugs) (see Workman and Collins, 2010); and, more recently, modulation by thalidomide analogs of the CRBN-CUL4 E3 ubiquitin ligase complex (Fischer et al., 2014). But, despite excellent progress, there are serious issues with the use of chemical probes in biomedical research.

Earlier commentaries and reviews in the chemical biology literature outlined the ideal attributes of “fitness factors” of high-quality chemical probes to be used to answer important biological and biomedical questions (see Frye, 2010, Workman and Collins, 2010). Recent articles, from both industrial and academic authors, highlight an increasing need for more and better quality chemical probes that can be applied, alongside an appropriate suite of biological reagents, to enable discoveries about fundamental biology and disease pathophysiology, and to thoroughly validate the roles of potential biological targets emerging from a range of approaches, including hypothesis-driven, screening-based, genomic, or disease-directed studies (Bunnage et al., 2013, Blagg and Workman, 2014, Garbaccio and Parnee, 2016). Academia and open-source initiatives are contributing to interest and efforts in this area by becoming more engaged in drug discovery, notably through screening chemical libraries against novel biological targets to spark the discovery of innovative chemical probes with the objective of building further understanding of target-based molecular pharmacology (Frye et al., 2011, Frearson and Wyatt, 2010). Importantly, evolving opinion and practice on the desired attributes of such chemical probes has highlighted both the promise and the peril of their use in biomedical research (Arrowsmith et al., 2015).

Why another opinion piece, why now, and why in this biological journal? A major concern, especially among researchers in the chemical biology and drug discovery communities, is that there is continuing evidence of the inappropriate use and promulgation of poor-quality chemical probes, perpetuating a worrisome and misleading pollution of the scientific literature, despite the best self-corrective efforts of practising experts and scientific journals, and notwithstanding increasing endeavors to inform the broader scientific community through primary publications, reviews, and conference sessions (Arrowsmith et al., 2015). An impetus for this article was the 2015 Annual Meeting of the American Association for Cancer Research (AACR) that hosted a number of plenary and educational sessions highlighting the importance of, and necessity for, high-quality chemical probes to further our understanding of the aberrant cell biology that fuels cancer initiation and progression (American Association of Cancer Research, 2015). While the lectures were well attended, there was a common view among invited speakers and commentators from the floor that such sessions are largely preaching to the choir and failing to connect to a really critical audience: namely, the wider cancer biology community who rely upon small-molecule tool compounds, often in harness with biological reagents, to interrogate cancer cell biology and who frequently draw important and highly impactful biological interpretations, whether correct or misleading, from such studies (Blagg and Workman, 2014).

Reviews and guidelines concerning chemical probes are usually published in specialist chemical biology journals for which the readership mainly comprises experts already well aware of the issues and current best practice (Frye, 2010, Workman and Collins, 2010, Bunnage et al., 2013, Blagg and Workman, 2014, Garbaccio and Parnee, 2016, Arrowsmith et al., 2015). We have written this article specifically for a general cancer biology audience (incorporating a glossary of technical terms) in an effort to reach out to this important and highly influential research community and to underscore why thorough characterization of both chemical and biological tools is so critical to the advancement of a biological understanding of cancer (and other diseases), as well as for robust target validation applied to drug discovery in both industry and academia. We provide a convenient “Dos and Don'ts” guidance for the selection and use of chemical probes in biological studies. We warn that selecting, as is common practice, your chemical probe from vendor catalogs that are inevitably driven by commercial considerations and lack scientific detail and expert opinion, or based on search engines whose results are biased in favor of what biologists have used in the past, will commonly not provide the most appropriate high-quality small-molecule tool compound to robustly test your biological hypothesis (Arrowsmith et al., 2015). In addition, we advise that good chemical probes will rarely emerge directly from a compound library screen; hit compounds from such screens commonly require extensive chemical optimization and biological profiling to generate high-quality chemical probes. Later, we will describe selected examples where erroneous use of poor-quality compounds has led to misleading or incorrect conclusions.

### Small Molecules Are from Mars, Biological Tools Are from Venus

Multiple authors have encouraged the use of small-molecule chemical probes alongside biological reagents to assess the importance of biological targets to various disease-relevant phenotypes, including cancer (e.g., Bunnage et al., 2013, Blagg and Workman, 2014, Garbaccio and Parnee, 2016). Paramount in their reasoning is that the widely used biological RNAi and clustered regularly interspaced short palindromic repeat (CRISPR) reagents commonly remove the protein in its entirety, whereas most good-quality small-molecule probes selectively modulate protein function without altering protein levels, thereby enabling interrogation of both the concentration- and time-dependent response (Blagg and Workman, 2014). Note, however, that small-molecule binding can sometimes cause degradation of the protein target, as shown for several kinase inhibitors, although such degradation may be slower than the rapid inhibition of, say, kinase signaling (Polier et al., 2013). Nevertheless, target protein levels should be monitored.

Although small-molecule and biological tool approaches are two different worlds in the same universe, it is interesting to see that these worlds are now converging with the advent, on the one hand, of specific protein function modulation through CRISPR gene-editing technology (Doudna and Charpentier, 2014) and, on the other hand, of the selective removal of whole proteins through small-molecule-directed proteolysis targeting chimera (PROTAC) technology and Cereblon-targeted protein degradation (Bondeson et al., 2015, Winter et al., 2015). Fundamentally important to both worlds is an understanding of the target selectivity of both small-molecule tools and biological reagents.

As contextual insight, we present and illustrate here an important but infrequently articulated perspective: namely that it is to be expected that most small molecules will interact with multiple biological targets when exposed to complex biological systems, including both healthy cells/organisms and aberrantly wired cancer cells/mouse models. By contrast, many optimized biological reagents, for example siRNA oligonucleotides and even more selective antibodies, are intrinsically more likely than small molecules to preferentially bind to their intended biological target, by virtue of the increased breadth, complexity, and, hence, specificity of their combined intermolecular interactions (Hann et al., 2001).

### Fragments to Fabs: Lessons in the Selectivity of Chemical Probes and Biological Tools

**Figure 1:**
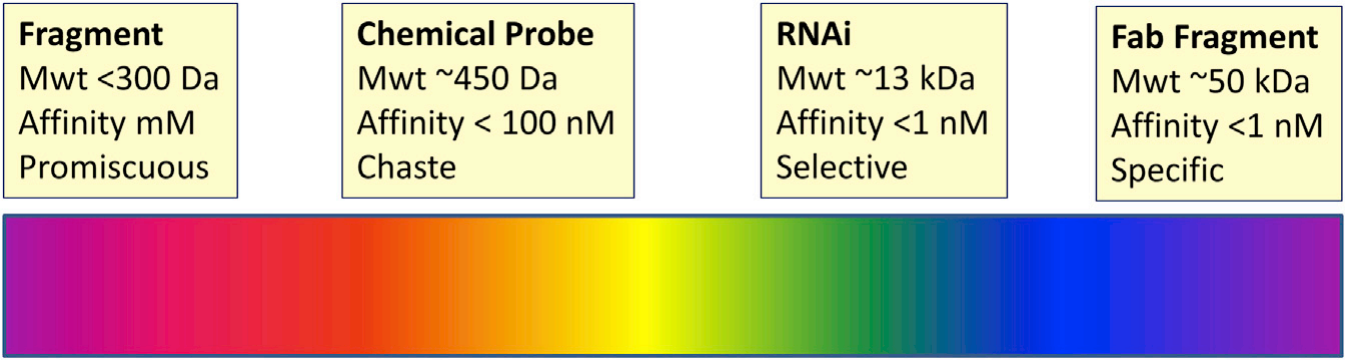
Fragments to Fabs: an Affinity-Selectivity Spectrum Increasing molecular weight increases the likelihood of specificity. Careful chemical optimization and biological testing must be carried out to minimize the risk of off-target effects in chemical probes.

The fragment-based approach to drug discovery, now often-lauded by medicinal chemists (Baker, 2013), is based upon the principle that low-molecular-weight fragments (those with MW < 300 Da and <15 heavy atoms: C, N, O, S, halogen, and not H) are very effective in sampling the theoretically available chemical space within their molecular weight range. By this we mean that a library comprising several thousands of fragment-like compounds effectively represents all the possible fragment-like molecules that could theoretically exist. The larger the number of heavy atoms, the greater the number of molecules required to exemplify all the theoretically possible combinations (Blum and Reymond, 2009). In addition, the lower molecular complexity (Bottcher, 2016) of fragment-like compounds offers a higher probability of the fragment matching the requirements of a particular protein pocket. Notably, however, fragments are commonly observed to exhibit weak binding affinities (0.1–1 mM) across diverse binding sites, provided that the biochemical assay methods are sufficiently sensitive to detect them (Figure 1) (Baker, 2013, Hall et al., 2014, Parker et al., 2017). This finding is consistent with the notion that complex, larger molecules are more likely to have unfavorable clashes with a given binding site (Hann et al., 2001, Leach and Hann, 2011). By analogy, small cars will fit inside most garages, while larger cars are more discriminating. Thus, fragments are not themselves chemical tools but are valuable starting points for structure-based design.

Compared with fragments, chemical tools and lead-like molecules generally encompass a higher molecular weight range (300–500 Da) and molecular complexity, thereby offering the opportunity to design stronger affinity to a target protein by optimizing ligand features to fit those of the chosen protein, known as “pharmacophore matching” (see Box 1) (Hann and Keserü, 2012). Biological reagents extend further along this size/complexity/selectivity spectrum (Figure 1). For example, optimized siRNA tools are of high molecular weight (average of 13.3 kDa for a 21 base-pair duplex siRNA), of generally high affinity (K_D_ < 1 nM for the binding of oligodeoxynucleotides to complementary mRNA) (Walton et al., 2001), and selective (rather than specific) by virtue of Watson-Crick base-pairing. Thus, a single mismatch in an antisense oligonucleotide can lead to a 500-fold affinity loss consistent with high target selectivity; nevertheless high concentrations of homologous mRNAs can still result in off-target effects (Hall, 2004).

Box 1 **Glossary of Terms**

**Activity-based protein profiling:** unbiased method to detect the diversity of proteins bound by a small molecule in a cellular environment by quantifying the displacement of a tagged ligand that binds to the active site of multiple, and preferably all, proteins in a biological class (Willems et al., 2014).
**Affinity:** describes the ability of a ligand to bind to a biomolecule. High-affinity compounds will occupy all available binding sites at low concentrations.
**Amphiphilic:** possessing both hydrophilic and lipophilic properties, for example, a soap or detergent.
**Electrophilic:** from the Greek “electron-friendly”; a molecule that attracts electrons or electron-rich species such as thiol-containing residues in proteins.
**Electrophilic warhead:** a chemically reactive group, such as an aldehyde, ketone, or unsaturated carbonyl compound, which is susceptible to reaction, most commonly with cysteine thiols (Blagg, 2010).
**Fragment-like molecule:** small molecules of low molecular weight, commonly defined as <300 Da, with less than 15 heavy atoms (C, N, O, S, and halogen).
**Hydrophilic:** from the Greek “water-friendly”; prefers an aqueous over a lipid environment.
**Lead-like molecule:** commonly defined as small molecules in the molecular weight range >300 Da and <500 Da.
**Ligand:** a substance, usually a small molecule, that binds to a biomolecule, commonly into a defined cavity or groove on a protein.
**Lipophilic:** from the Greek “fat-friendly”; prefers a lipid over an aqueous environment.
**Molecular complexity:** a context-dependent concept used by diverse scientific communities that is difficult to define and quantify (Bottcher, 2016). For the purposes of this perspective, it is the number of features in a molecule that can potentially interact with a biological target (Hann et al., 2001).
**Pharmacophore:** a description of the features of a molecule that are necessary for recognition by a biological target.
**Pharmacophore crossing:** the propensity for ligand structural features to be complementary to binding sites in multiple protein targets.
**Pharmacophore matching:** complementarity of the ligand pharmacophore to the features of a biological target.
**Phenotypic assay:** screening of compounds in cellular or animal models of disease to identify molecules that cause a desirable change in the phenotype (Swinney and Anthony, 2011).
**Potency:** describes the amount of compound required to elicit a biological effect. Potent compounds will elicit an effect at low concentrations. IC_50_ is defined as the half-maximal inhibitory concentration.
**Reactive metabolite:** electrophilic species generated by metabolism of a parent molecule. Reactive metabolites can bind covalently to biological macromolecules, such as proteins and DNA, thereby affecting their function.
**Selectivity:** demonstration of selective affinity for the target protein of interest versus other members of the protein family and selected members of other protein families.
**Stereoisomeric:** molecules that have the same molecular formula and connection of bonded atoms, but differ in the three-dimensional orientation of their atoms in space.
**Target engagement:** quantitation of the binding of a small-molecule ligand to its target protein(s) in a biological system, such as a cell or animal model, by biophysical methods or displacement of a tracer ligand.
**Target modulation:** measurement of proximal downstream biological sequelae (Banerji and Workman, 2016).

Antibody tools provide the opportunity for still further optimization of target binding affinity with concomitant target specificity; the fragment antigen-binding (Fab fragment) region on an optimized antibody binds to antigens with sub-nM affinity and high specificity (de Haard et al., 1999). Interestingly, although the need for characterization of antibody affinity and selectivity may be better recognized by biologists than is the case for chemical probes, the validation of antibody reagents is often also inadequate (Bordeaux et al., 2010, Ponten et al., 2008, Roncador et al., 2016, Uhlen et al., 2016). The US National Cancer Institute Antibody Portal and EuroMabNet serve as publicly available community resources for unbiased antibody validation, including demonstration of selective binding (National Cancer Institute, 2017, European Monoclonal Antibodies Network, 2017). Although there is extensive contamination of the literature with poor-quality antibodies and also poorly controlled siRNA studies, and certainly scope for improvement and implementation of best practice, we nevertheless observe that biologists are less aware of the potential pitfalls with chemical probes, including a lack of recognition that un-optimized small-molecule reagents have inherently poor selectivity and hence should be picked with even greater caution (Figure 1).

This spectrum of affinity and selectivity in relation to molecular weight provides an informative context for consideration of small-molecule chemical tools which, with appropriate molecular design and control molecules, have the potential to be as sufficiently selective and informative as optimized biological reagents. However, as highlighted earlier, without informed medicinal chemistry optimization, promiscuity should be regarded as a probable scenario. Thus, high-quality antibody reagents can be expected to bind with high affinity and specificity for their intended target in *in vitro* cell-based assays or *in vivo* animal models, whereas it is highly likely that non-optimized small molecules, even though they may appear selective across *in vitro* biochemical screening panels, are likely to bind to multiple unintended biological targets in the context of biological systems. This is especially worthy of consideration when contemplating a screen of diverse chemical entities in a cell-based phenotypic assay (Box 1) where numerous false-positive hits are more likely to be observed compared with a simpler biochemical screen with one or a few recombinant proteins. In this context, both smart design of the cell-based assay cascade and the quality of the chemical library are critical to enable subsequent optimization of identified hits to inform deconvolution of their biological targets (Blagg and Workman, 2014, Dale et al., 2015).

### Complementarity of Biological and Chemical Tools

While recognizing the need to critically assess the selectivity and effectiveness of chemical tools, the use of well-designed orthogonal studies employing biological approaches can provide further confidence in the mechanistic specificity of the resulting phenotypes (Fu et al., 2014, Cho et al., 2016). Importantly, to get the best out of chemical probes requires a critical evaluation of their quality, suitability, and selectivity for the particular biological hypothesis under scrutiny; for example, by testing for compound binding to biological targets likely to elicit a similar phenotypic response. Biologists need to question the acceptability and credibility of chemical probes just as much as, and indeed more so than, any biological reagents they use. A gold-standard test to validate the functional response to a chemical probe is to demonstrate reversal of compound-induced biological effects in the presence of a mutation that abrogates compound binding to the biological target (Kaelin, 2017, Kasap et al., 2014). Another valuable technique is the engineering of a functional target to interact with chemical probes not recognized by the wild-type protein (Bishop et al., 2000, Baud et al., 2014). An additional approach is to determine the effects of the chemical probe in cells where the putative biochemical target has been removed by CRISPR-Cas9. The value of this methodology was recently exemplified by the devalidation of the proposed oncoprotein maternal embryonic leucine zipper kinase (MELK): thus CRISPR-Cas9 deletion of MELK was tolerated in a range of cancer cell lines, and the clinical candidate MELK inhibitor OTS167 retained activity in MELK-knockout lines, indicating that the antiproliferative activity of this drug is mediated via an off-target mechanism (Lin et al., 2017).

Whereas the onus frequently rests on the originating research team to demonstrate the specificity of a given biological tool for its intended target over relatively few closely homologous proteins, it is often the purview of the medicinal chemist to prove, to the best of their ability and often with limited budget, that a small-molecule ligand is not overtly promiscuous across the entire druggable proteome, which is encoded by up to 7,668 genes comprising the “druggable genome” (Griffith et al., 2013, Hopkins and Groom, 2002, Overington et al., 2006, Santos et al., 2017). To limit the scope of selectivity investigation to a realistically testable scale, it is commonly assumed that the off-targets of a chemical probe will relate to the primary target by protein sequence and folding architecture of the secondary structure. Indeed, protein families that share the same endogenous ligand (see Box 1) or co-factor binding sites are more likely to bind ligands that mimic the respective endogenous molecule. However, it is now clear that many small-molecule ligands bind to unrelated proteins from quite different families, and detailed investigations do not support a simple “code” with which to predict all such off-target ligand-binding sites from protein sequence or structure alone (Barelier et al., 2015, Lounkine et al., 2012). Given the diversity of druggable proteins (Griffith et al., 2013, Hopkins and Groom, 2002, Overington et al., 2006, Santos et al., 2017), and recent data demonstrating the promiscuous binding of fragment molecules (see above) to hitherto undrugged proteins (Parker et al., 2017), we recommend taking a routinely cautious and skeptical approach before investing significant resource in using a chemical probe to test important biological hypotheses in labyrinthine cellular and organismal systems.

### Selectivity of Chemical Probes versus Drugs

Underpinning the above warning of caution in the selection of chemical probes for biological studies is the realization that an un-optimized small molecule is unlikely, until proven otherwise, to demonstrate high-level specificity for its intended biological target. Perhaps surprisingly to many biologists, this lack of selectivity also applies to many marketed drugs. For example, in a study of 392 oral drugs tested versus an average of 7.3 unique biological targets, more than half had 50% inhibitory (IC_50_) values of less than 1 μM for two or more targets, suggesting significant promiscuity in drugs (Gleeson et al., 2011). A consortium of four pharmaceutical companies published a valuable large-scale analysis of their combined *in vitro* profiling of marketed drugs versus broad panels of biological targets associated with adverse clinical outcomes. This demonstrated that approximately 30% of marketed drugs tested at 10 μM (a screening concentration consistent with the upper limits of recommended cell-based assay concentrations, see next section) show >50% inhibition of >5% of biological targets tested (Bowes et al., 2012). While the presence of such significant secondary pharmacology in a drug is often acceptable, and even desirable to elicit the required therapeutic efficacy and mitigate resistance, it is potentially confounding for the purpose of interrogating a specific biological target in the context of a detailed mechanism-based biological investigation (Box 2). Consequently, we suggest that a critical, weight-of-evidence argument is necessary to build confidence in the application and subsequent interpretation of data generated with chemical probes including, importantly, the powerful use of more than one, and ideally multiple, chemical probes of differing off-target profiles and derived from diverse chemical classes, along with appropriate structurally matched inactive or less-active control analogs, as well as complementary use of the biological and genetic approaches mentioned earlier, particularly rescue experiments.

Box 2 **Selectivity Profile of Chemical Probes versus Drugs**

**Chemical probe: used to examine the biological hypothesis and selective with respect to:**
• Proteins whose altered function could confound the interpretation of the biological hypothesis under test, e.g., other cell-cycle kinases when testing a specific cell-cycle mechanism
• Highly homologous proteins
• Protein families that share the same endogenous ligand (Box 1) or co-factor binding sites

**Drug: used to build confidence in a safe therapeutic window and sufficiently selective with respect to:**
• Proteins known to elicit an important short-term adverse clinical outcome, e.g., Herg inhibition and torsade de pointes (Hancox et al., 2008)
• Proteins where there is a risk of long-term adverse clinical outcomes, e.g., 5HT2B agonism inducing cardiac valvulopathy (Rothman et al., 2000)
• Proteins known to elicit unfavorable drug-drug interactions, e.g., cytochrome P450 family proteins

### The Pharmacologic Audit Trail

**Figure 2:**
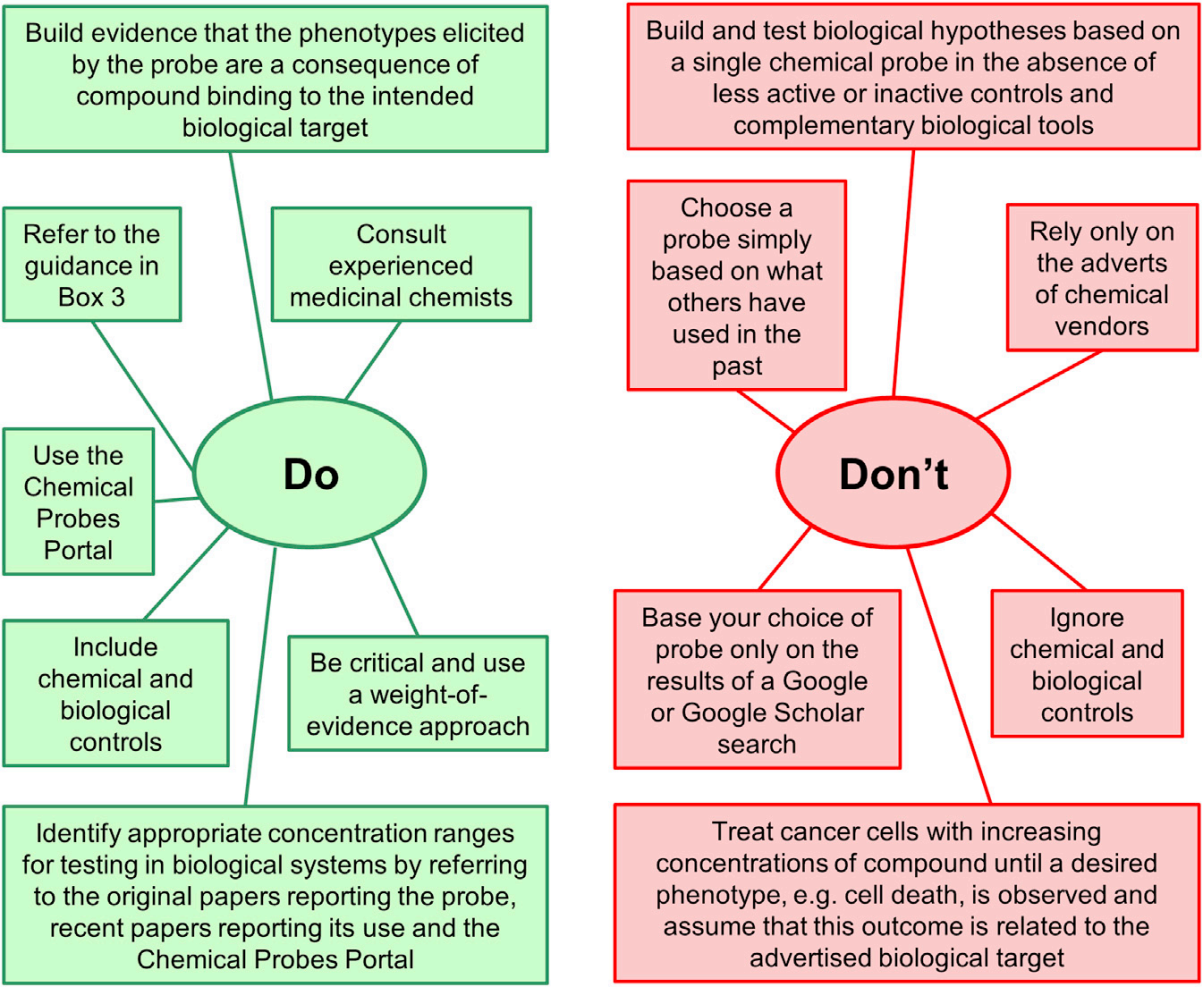
Dos and Don'ts of Chemical Probes

In addition to cell-free biochemical data demonstrating appropriate potency and selectivity (Workman and Collins, 2010), it is also important to obtain robust evidence of on-target activity, and ideally also off-target effects, of chemical probes in cellular and organismal models. This is consistent with the concept we have codified and promulgated of the Pharmacologic Audit Trail (Banerji and Workman, 2016), and is also one of the four pillars of cell-based target validation using chemical probes described by Bunnage et al. (2013). Unfortunately, it is still common practice to treat cancer cells with increasing concentrations of compound until a phenotype, commonly cell death, is observed. Compound promiscuity is concentration-dependent, thus the higher the concentration of compound applied, the more likely the observed outcome will be due to off-target pharmacology, and results obtained from testing compounds in cancer cells at concentrations above 10–20 μM should be treated with caution. Rather than assuming that the observed phenotypic consequence is driven by the intended biological target, it is critically important to obtain evidence for concentration-dependent and selective modulation of the intended molecular target at exposures that make sense with respect to the observed phenotypic outcomes (Figure 2 and Box 3).

Box 3 **Factors that Determine the Fitness and Quality of Chemical Probes**

**Chemical structure**
• Absence of chemically reactive groups (structural alerts) and/or Pan-Assay Interference Compounds (PAINS) that may elicit off-target pharmacology or interference with proposed assay methodologies.
• Absence of chemically unstable and/or metabolically unstable moieties that may compromise the interpretation of cell-based or *in vivo* animal studies.
• Membrane permeability and solubility consistent with cell penetration.

**Potency (biochemical)**
• Demonstration of sufficiently potent binding affinity to the biological target of interest in multiple orthogonal assays; IC_50_ is defined as the half-maximal inhibitory concentration.

**Selectivity (biochemical)**
• Demonstration of sufficiently selective affinity for the target protein versus other members of its protein family as well as selected members of other protein families (e.g., by screening in broad ligand-binding assays).

**Selectivity (cell-based)**
• Evidence for concentration-dependent and selective modulation of the desired biochemical target in cells at concentrations that are interpretable in the context of *in vitro* potency.

**Selectivity (*in vivo*)**
• Evidence for on-target biomarker modulation at *in vivo* exposures consistent with maintenance of selective pharmacology.

**Availability of appropriate controls**
• Provision of a less-active or inactive chemical tool with a similar chemical structure and properties to the active chemical probe.
• Provision of a second chemical probe from a structurally distinct chemical series.

There are now a range of cell-based technologies for measuring immediate target engagement (Box 1) by chemical probes. These include direct-binding chemical proteomics technologies such as activity-based protein profiling (ABPP) (see Box 1, Willems et al., 2014), spatial proximity methods such as fluorescence resonance energy transfer, bioluminescence resonance energy transfer, cellular thermal shift assays, and fluorescence polarization microscopy, in combination with companion imaging probes (Simon et al., 2013, Huber, 2017). Similarly, it is also important to determine the resulting downstream effects of chemical probes on biochemical pathways and processes by assessing a range of mechanism-based pharmacodynamic biomarkers, preferably quantitatively (Banerji and Workman, 2016). Use of more than one biomarker can be helpful to build confidence in an on-target mechanism of action. Biomarkers, such as proteins, that exhibit an increase in expression in response to the exposure of cells to treatment with a chemical probe can be valuable, since such an increase is more likely to be the result of compound-specific action than a consequence of the widespread protein degradation that occurs during cell death (see also Kaelin, 2017).

Unbiased determination of the consequences of direct target engagement and the resulting target modulation (Box 1) in cells and organisms can be addressed using technologies such as transcriptional and (phospho)proteomic profiling, for which valuable resources and interrogable knowledge-bases are becoming freely available to help inform on mechanism of action (Lamb et al., 2006, Schenone et al., 2013, Seashore-Ludlow et al., 2015, Muroi et al., 2010); these can reveal both on- and off-target effects. In addition, extensive public resources are available describing the known effects of chemical compounds, including drugs and chemical probes, as well as genetic perturbation, on the viability or proliferative potential of large panels of genetically annotated human cancer lines (Cancer Cell Line Encyclopedia Consortium and The Genomics of Drug Sensitivity in Cancer Consortium, 2015, Seashore-Ludlow et al., 2015). Importantly, it should be noted that a highly target-specific chemical probe may nevertheless beget pleiotropic downstream biological consequences if its biochemical target is nodal to multiple biological pathways; thus, target specificity does not always beget biological specificity (Dale et al., 2015).

In the following sections we highlight and discuss supporting evidence for the ubiquity of small-molecule promiscuity. We illustrate this critical point with appropriate examples to caution biological researchers in the best-practice use of chemical probes and to arm them with pertinent questions that we believe it is essential to ask before embarking upon intricate, time-consuming, and expensive biological studies with chemical tools that may be risky or even fatally flawed (Figure 2 and Box 3). Representative examples are portrayed from cancer biology, including our own experience, to illustrate how poorly characterized chemical probes can promote and perpetuate inappropriate biological conclusions. We emphasize the warning “caveat emptor” – let the buyer beware (Arrowsmith et al., 2015) – and seek to equip the biological researcher with advice to avoid investment in the equivalent of a defective global positioning (or satellite navigation) system.

### MTH1 as a Cancer Target?

**Figure 3:**
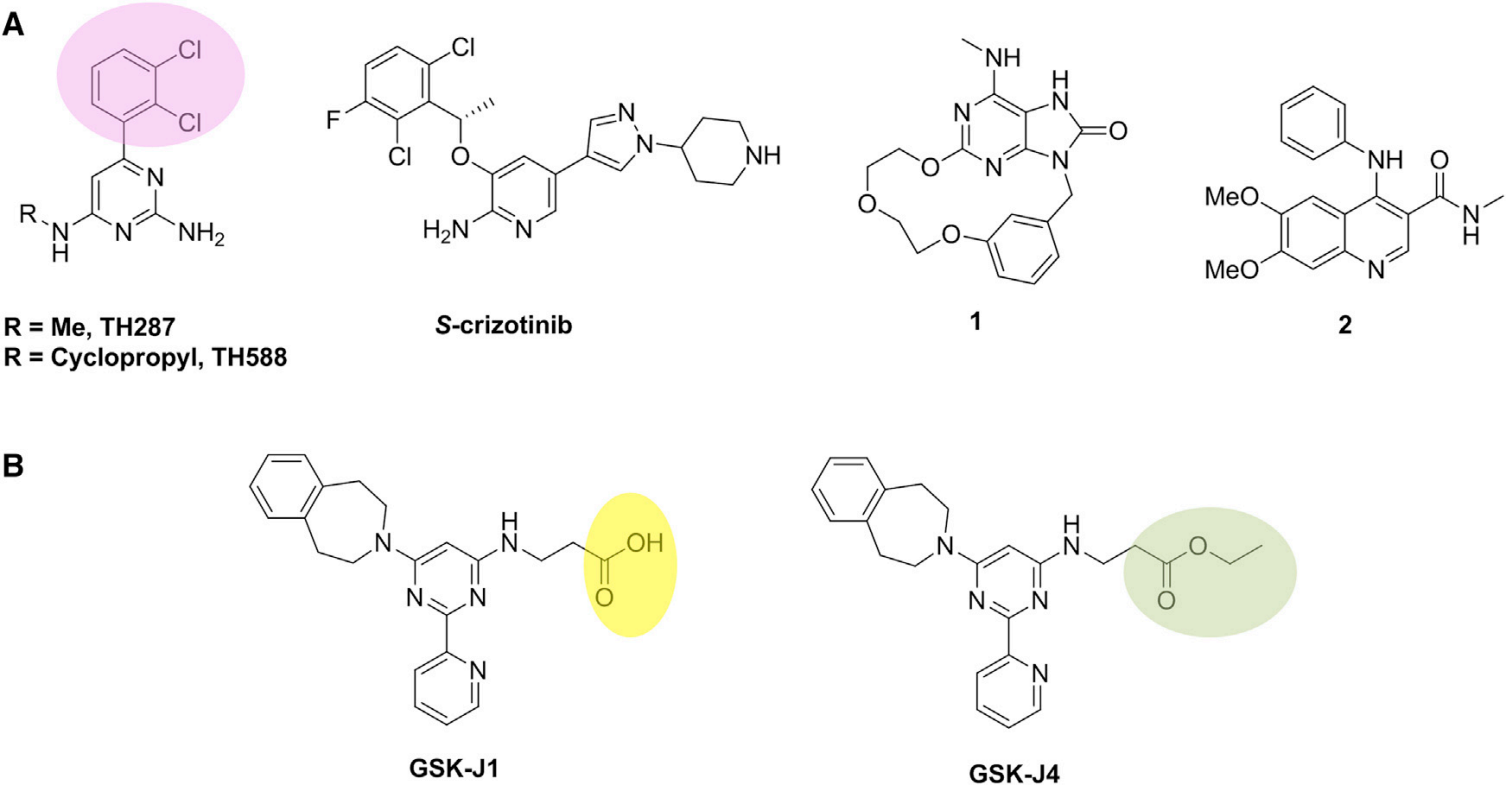
MTH1 and Histone Demethylase Chemical Probes (A) MTH1 ligands TH287 and TH588 with lipophilic moiety highlighted in lilac, *S*-crizotinib, and characterized chemical probes **1** and **2**. (B) Histone demethylase inhibitor GSK-J1 with the highly ionized and poorly cell-penetrant carboxylic acid moiety highlighted in yellow and its corresponding cell-penetrant ethyl ester GSK-J4 (ester moiety highlighted in green).

A recent study elegantly highlights the complementary value and importance of using orthogonal chemical and biological approaches to target validation in cancer (Kettle et al., 2016). Comparison of the effect of multiple high-quality chemical probes for the DNA damage repair enzyme MTH1 with that of siRNA and CRISPR biological tools demonstrated that, in marked contrast to previous reports, neither chemical inhibition of MTH1 activity nor RNAi and CRISPR-mediated knockdown of MTH1 elicited killing of cancer cells. Previously reported chemical probes for MTH1 include TH287 and TH588, as well as S-crizotinib (Figure 3A). Pharmacophore crossing (see below and Box 1) of toll-like receptor 7 (TLR-7) ligands with the MTH1 target facilitated the discovery of potent and selective MTH1 chemical probes **1** and **2**, both devoid of off-target activity in broad kinase or secondary pharmacology profiling, and with clear evidence for MTH1 binding in cells demonstrated by cellular thermal shift profiling. In contrast to TH287 and more “water-hating” lipophilic analogs (see below and Box 1), neither compound **1** nor **2** demonstrated antiproliferative activity across a panel of human cancer cell lines, nor did they show evidence for the expected modulation of the DNA damage response or apoptotic biomarkers. Although the later authors replicated the original reports of MTH1-dependence by siRNA-mediated knockdown using the same siRNA reagents, further exploration with additional siRNA reagents targeting MTH1, and also with CRISPR-mediated silencing of *MTH1*, failed to confirm the original findings, strongly suggesting off-target activity with the original siRNA reagent. Finally, in a critical experiment, both TH287 and *S*-crizotinib killed MTH1-null cancer cell lines, thus implicating off-target-mediated pharmacology. Subsequent proteomic profiling of TH287 and TH588 indicated that tubulin-binding is responsible for their cytotoxic effects (Kawamura et al., 2016). Taken together these comprehensive studies clearly illustrate the potential risks of off-target pharmacology with both small-molecule chemical tools, particularly compounds with high lipophilicity (see below for further discussion of this concept and also Box 1), and RNAi reagents as well (Persengiev et al., 2004). They also highlight the important need to use several high-quality chemical and orthogonal biological tools in parallel during target validation, in order to reach robust conclusions. This exemplar case history also serves to highlight that original results, while sometimes reproducible, can nevertheless lead to incorrect conclusions. Examples of lack of robustness such as this almost certainly account for some of the discouraging results when biologists and drug discovery scientists attempt to replicate and extend original findings during detailed biological target validation studies, prior to significant resource investment (Prinz et al., 2011, Begley and Ellis, 2012, Kaelin, 2017).

### Targeting Multi-Domain Proteins: SMARCA2, HIF-2α, and the JmjC-Containing Histone Lysine Demethylases

In some cases, biological tools provide insufficient information on the role of a particular functional protein domain. For example, the SWItch/Sucrose Non-Fermentable (SWI/SNF) complex modulates chromatin structure via two mutually exclusive catalytic subunits, SMARCA2 and SMARCA4, both of which possess a bromodomain and a catalytic ATPase domain. An elegant suite of studies carried out using biological and genetic experiments, as well as a complementary fit-for-purpose chemical probe for the SMARCA2 bromodomain, demonstrated that knockdown of the entire SMARCA2 protein elicits an antiproliferative phenotype in clinically relevant SMARCA4-mutant tumors. This synthetic lethality was rescued by a bromodomain-mutant form but not by an ATPase-dead SMARCA2 protein. Furthermore, synthetic lethality was not observed by pharmacologic inhibition of bromodomain function using the selective chemical probe PFI-3. Taken together these results demonstrate that the ATPase activity of SMARCA2 is required for maintaining tumor cell proliferation, whereas the bromodomain function is not (Vangamudi et al., 2015). They also highlight the importance of drugging the more challenging ATPase domain to achieve pharmacological synthetic lethality.

Also illustrative of such a comprehensive approach is an exemplary recent study of PT2399, a small-molecule antagonist of the PAS-B protein-binding domain of the multi-domain helix-loop-helix (bHLH)-Per/Arnt/Sim (PAS) transcription factor HIF-2α. This study used PT2399 in parallel with a suite of biological tools, including an HIF-2α-mutant protein shown to block PT2399 binding to the PAS-B domain, to demonstrate that PT2399 decreases HIF-dependent transcription and antiproliferative activity in an on-target HIF-2α-dependent manner through binding to the PAS-B domain, and also that the differential therapeutic sensitivity of human kidney cancer cell lines to PT2399 likely reflects differences in their HIF-2α dependence and indicates the need for predictive biomarkers (Cho et al., 2016).

Significant effort has been applied to the discovery of small-molecule chemical tools for the JmjC-containing family of histone lysine demethylases (KDMs). Such tools are critical if we are to understand the importance of JmjC-domain histone demethylase catalytic activity (as distinct from the chromatin localization or scaffolding functions of full-length KDM proteins) in determining a given biological outcome. As described above, such a distinction cannot be made using RNAi-mediated protein knockdown studies that take out the whole protein, unless more technically demanding rescue studies are carried out with appropriate domain-targeted mutants. For the KDM4 and KDM5 proteins, elegant biochemical studies using peptide and nucleosome substrates have shown that their Tudor domains act as co-operative chromatin-homing motifs to both direct and enhance the rate of JmjC-mediated demethylation of adjacent histone methyl marks (Ortiz Torres et al., 2015, Su et al., 2016). Increasing evidence for the role of multiple members of the KDM protein family in the initiation and maintenance of cancer has fueled mounting interest in these targets (Højfeldt et al., 2014). Details of a number of chemical tools that modulate the catalytic activity of KDM proteins have been published (McAllister et al., 2016); however, many of these tools, such as GSK-J1, suffer from low cell permeability associated with the presence of a highly ionized carboxylic acid moiety. Without robust evidence of target modulation (Box 1) with such compounds, linking observed cell-based phenotypes to inhibition of demethylase activity is dangerous. To overcome the expected poor cell permeability of inhibitors bearing a charged carboxylic acid, the corresponding ester “prodrugs” have been investigated, notably GSK-J4 which is the cell-permeable ester ‘prodrug’ of the KDM6-selective inhibitor GSK-J1 and releases the active agent through hydrolysis by cellular esterases (Figure 3B). Exposure of cells to GSK-J4 led to the expected increase of nuclear H3K27me3 indicating cell-based target inhibition, whereas neither GSK-J1 nor a structurally matched inactive control ester had this effect (Kruidenier et al., 2012). These findings clarify the previous ambiguity concerning the catalytic function of H3K27-specific Jmjs in regulating inflammatory responses. This represents a valuable approach to discovering selective and cell-permeable chemical probes, and potentially drugs, for KDMs.

### Notable Characteristics of Poor-Quality Chemical Probes

**Figure 4:**
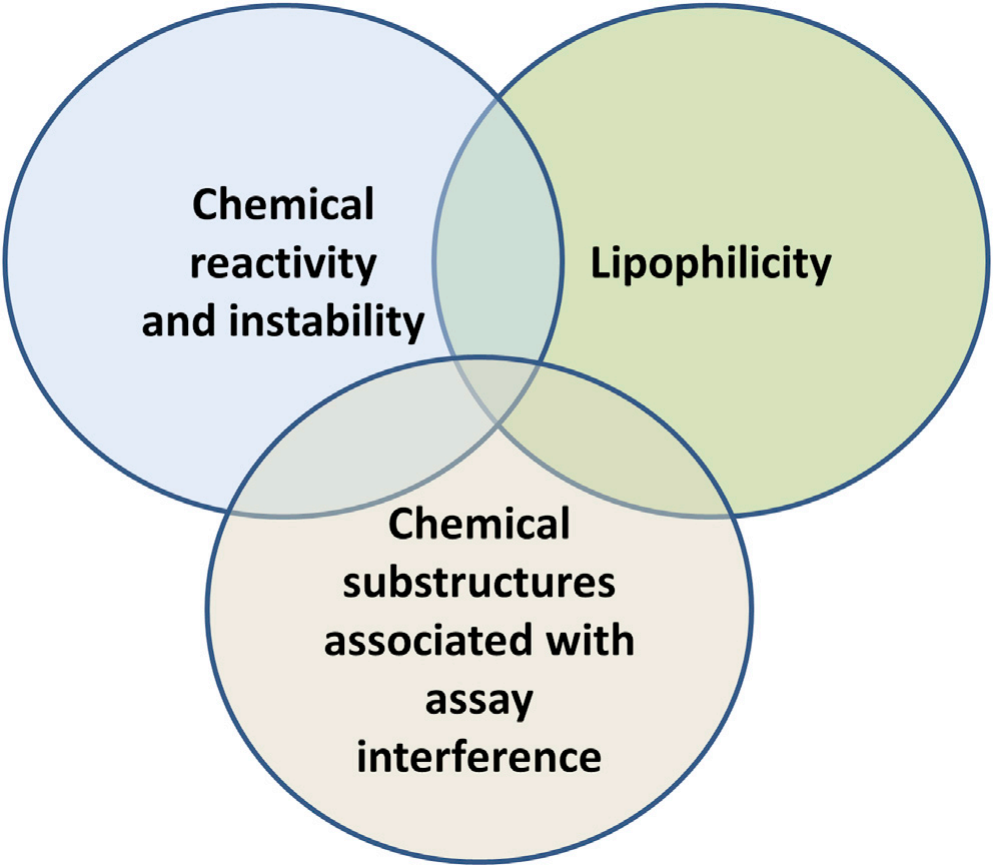
Factors Likely to Influence Promiscuity in a Claimed Chemical Probe (1) Chemical reactivity and instability (a chemical structure that is reactive or unstable in the medium of a biological assay); (2) lipophilicity (the propensity for a small molecule to leave the aqueous environment and bind to proteins irrespective of the protein structure or sequence); (3) chemical substructures associated with assay interference (Pan-Assay Interference Compounds, PAINS, see text). The overlap in the Venn diagram indicates that more than one undesirable feature can occur in a given chemical structure which magnifies the likelihood of problems occurring in biological use.

In this section we illustrate some of the undesirable properties associated with chemical tools of limited utility and lessons that can be learned from their use (Figure 4).

#### Not What It Says on the Tin

Unfortunately, there are multiple documented cases where the experimentally determined chemical structure of the purchased material is inconsistent with the label description. For example, the widely used phospholipase and sphingomyelinase inhibitor D609 has eight possible stereoisomeric forms, all of which present a different potential protein-binding pharmacophore. A recent study (Kato et al., 2016) described the synthesis of all of these isomers and demonstrated their differing *in vitro* inhibition potencies versus phosphatidylcholine phospholipase C. Furthermore, the authors discovered that commercial vendors provide differing isomeric mixtures of the compound. More worrying still, at least 18 commercial suppliers of the leukemia drug bosutinib, which inhibits the BCR-ABL tyrosine kinase with additional activity on Src-family tyrosine kinases, were found to be selling an incorrect structural isomer of the compound (Extance, 2015). This highlights the importance of verifying the chemical integrity of purchased probes, for example by obtaining appropriate chemical data characteristic of the molecular structure (commonly mass spectrometry and nuclear magnetic resonance profiles) through collaboration with chemistry colleagues, or a fee-for-service facility, and comparing the results with data in the literature that was generated from the authentic material – caveat emptor again (Arrowsmith et al., 2015).

**Figure 5:**
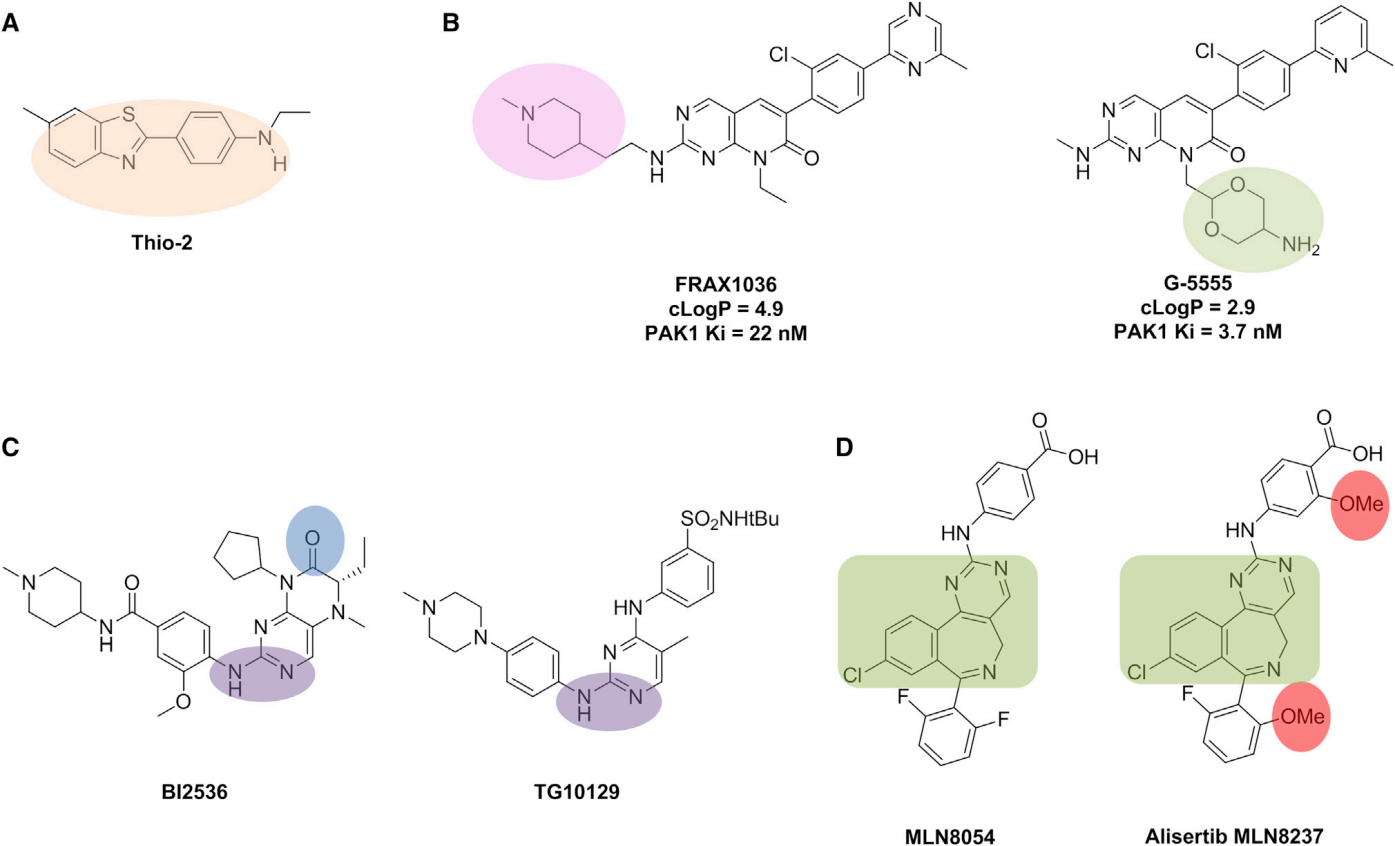
Thio-2, Lowering Lipophilicity and Pharmacophore Crossing (A) Chemical structure of Thio-2 with the reactive metabolite precursor highlighted in orange. (B) Lowering the calculated lipophilicity (cLogP) of the PAK1 inhibitor chemical tool G-5555 by 100-fold compared with starting compound FRAX1036 by removal of the lipophilic side chain (lilac) bearing a highly basic nitrogen and addition of a tolerated polar side chain (green) bearing a weakly basic nitrogen. (C) Pharmacophore crossing: the PLK1 kinase-binding motif of BI2536 is highlighted in purple and the BRD4 Asn140-binding motif is shaded blue. The kinase-binding motif responsible for both JAK2 and BRD4 affinity of TG10129 is shaded purple. (D) MLN8054 and the follow-on Aurora A candidate alisertib MLN8237; the benzodiazepine scaffold is highlighted in green; the structural differences in MLN8237 that abrogate GABA_A_ α-1 benzodiazepine binding are highlighted in red.

In a third example that links to the subsequent narrative, Thioflavin S has been reported to modulate the interaction of BAG-1 with the molecular chaperones HSC70 and HSP70, and also with CRAF, both *in vitro* and in human breast cancer cell lines (Sharp et al., 2009). Thioflavin S is a complex mixture of compounds and, originally, its biological activity was not definitively attributed to any one component; however, a subsequent study isolated and purified Thio-2 (Figure 5A), a component which retained the ability of Thioflavin S to block the BAG-1/HSC70 interaction (Enthammer et al., 2013). Despite this advance, the Thioflavin class of molecules, to which Thio-2 belongs, exhibit multiple sources of off-target activities, including CYP1A1-mediated generation of reactive intermediates and DNA-adduct formation (Chakraborty et al., 2010) as well as PAINS motifs (see next section), casting strong doubt upon the credentials of these compounds as high-quality chemical probes. Consultation with expert chemistry colleagues can avoid wasted time and effort.

A particular egregious behavior that must be eliminated is the publication of biological results for compounds where the chemical structure is not disclosed in the paper or included within a cited reference. This is absolutely unforgivable since it is impossible to interpret the biological findings without sight of the compound structure, nor can the results be independently verified. Reviewers and editors should be vigilant and resolute about abolishing this practice. It is not acceptable in a chemical journal and nor should it be in a biomedical one.

#### Ligand Promiscuity

Multiple factors are well known by chemical biologists and medicinal chemists, but commonly less so by academic biologists, to promote non-selective binding of small-molecule ligands to biomolecules. Notable among these “alerts” are chemical reactivity and instability, susceptibility to repetitively coupled reduction and oxidation (so-called redox cycling), chelation of essential metals, and lipophilicity (characterized by “lipid-loving” or “water-shy” behavior) (Bruns and Wilson, 2012, Peters et al., 2009, McGovern et al., 2003). The presence of more than one alert in a chemical structure magnifies the concern (Figure 4). These undesirable features, which commonly drive promiscuous binding in the context of high-throughput screening (HTS), lead to certain compounds being classed as “frequent-hitters” or even “chemical imposters” (Baell and Walters, 2014). The learning available from analysis of promiscuous HTS hits (Bruns and Wilson, 2012, Peters et al., 2009, McGovern et al., 2003, Baell and Walters, 2014, Baell and Holloway, 2010) is equally applicable and relevant to the characterization of chemical probes for biological use. The claimed description of a compound as a “probe” does not necessarily confer upon it better attributes than your average small molecule – caveat emptor once more.

#### Pan-Assay Interference Compounds

Often-cited Pan-Assay Interference Compounds (PAINS) are one subclass of potential rogue molecules that contain substructures that have been associated with an increased risk of interference with certain assay detection methods (Baell and Holloway, 2010). Importantly, despite the fact such compounds do not necessarily bind to multiple proteins, they may be viewed as promiscuous by virtue of their pan-assay interference. Biologists do need to be aware of the dangers of PAINS compounds. However, a recent publication highlights that computational PAINS filters may inappropriately flag some compound classes, noting the potential for incorrect prediction of promiscuity and reinforcing the need for orthogonal experimental assays to confirm (or otherwise) compound activity versus their intended primary biological target (Capuzzi et al., 2017). Some commentators have noted that access to multiple orthogonal assay formats may be limited in the academic setting (Lowe, 2017); however, such profiling of chemical probes (Boxes 2 and 3) is essential to mitigate the risk of significant further investment in flawed compounds. Multiple orthogonal assay formats have been developed and proven reliable for well-studied protein classes (e.g., kinases, G-protein coupled receptors and ion channels) (Janzen, 2014), and many are now available through fee-for-service vendors. Open innovation and crowdsourcing solutions, which enable the distributed evaluation of proposed chemical tools in multiple assays across the research community, are also to be welcomed (Structural Genomics Consortium, 2011). Further to this, provision of grant supplements for probe profiling would encourage best practice and be consistent with NIH requirements for the authentication of data and reagents in grant applications (Lauer, 2016).

#### Chemical Reactivity and Instability Beget Biological Promiscuity

Small molecules that are intrinsically chemically reactive, or that generate high concentrations of chemically reactive metabolites, are likely to bind covalently and indiscriminately to off-target proteins. A recent computational study demonstrates that frequent-hitter compounds in HTS exhibit increased chemical reactivity compared with selective compounds, mainly due to their electrophilic character (Curpăn et al., 2014). Such properties can be highlighted in the selection of chemical probes by applying appropriately validated *in silico* structural filters (Sushko et al., 2012), and also by seeking expert advice from medicinal chemists and chemical biologists.

On the other hand, there is a resurgence of interest in chemical probes that form a designed-in directed covalent interaction with their target protein, i.e., an initial rapid and reversible binding of the designed small molecule to a defined pocket on the target protein that is followed by formation of an irreversible covalent linkage. This is an attractive approach, particularly for the modulation of hitherto undruggable protein targets; key advantages include the ability to completely ablate a specific protein function without removing the protein, while at the same time avoiding the need for challenging pharmacokinetic optimzation as a result of essentially irreversible modification of the protein target. The directed covalent approach also benefits from an increased scholarship on electrophilic “warheads” (see Box 1) that, with an appropriately optimized scaffold, offer the potential to selectively bind to the protein of interest (Oprea et al., 2009, Liu et al., 2013). GSK-LSD1, a selective chemical probe for the histone lysine demethylase KDM1A (LSD1) (Structural Genomics Consortium, 2017), and the irreversible EGFR-mutant selective kinase inhibitor osimertinib (now approved in non-small-cell lung cancer), exemplify this approach (Finlay et al., 2014). There is a caution here, however, since application of ABPP using mass spectrometry-based proteomics (see Box 1) to elucidate the proteome-wide selectivity of covalent kinase inhibitors has illustrated the potential for extensive concentration-dependent off-target activity through covalent binding, particularly to exposed cysteine, but also potentially to exposed serine, threonine, or lysine residues across multiple diverse protein families (Lanning et al., 2014). For example, ibrutinib was shown to bind to multiple protein targets at concentrations previously used to attribute cellular phenotypic behavior specifically to BTK inhibition. In the context of cancer therapy, an additional and notable caution, irrespective of the aforementioned issues with chemically reactive inhibitors, is the risk of the emergence of resistant clones harboring mutations to the protein residues targeted by covalent modifiers (Engel et al., 2015).

#### Lipophilicity Begets Small-Molecule Aggregation and Biological Promiscuity

Lipophilic “grease-loving”, also called hydrophobic “water-hating”, molecules share a propensity to escape the aqueous environment (McGovern et al., 2003). At high concentrations in the buffer solutions used in biological assays such compounds commonly self-condense into aggregates or micelle-like bodies, which may present themselves as globular composites to proteins or cell membranes in biological systems. Molecules with greasy and polar groups at opposite ends (so-called “amphiphilic” molecules) are particularly prone to self-aggregation. Unsurprisingly, this phenomenon frequently leads to non-specific effects on multiple proteins and hence misleading observations unrelated to the pharmacology of the component small molecule (Blevitt et al., 2017, Irwin et al., 2015, Leeson and Springthorpe, 2007). Similarly, hydrophobic interactions between ligand and protein contribute significantly to favorable binding energies and, as a result, lipophilic molecules benefit from a greater driving force to leave the aqueous environment and enter into complementary lipid/protein environments, thereby resulting in multiple weak off-target interactions. Such interactions become more important at the high compound concentrations in aqueous media commonly used in biological assays (Tarcsay and Keserű, 2013, Peters et al., 2009). Lowering the lipophilicity of the PAK1 inhibitor chemical tool G-5555 by 100-fold compared with the starting compound FRAX1036 resulted in an improved on-target PAK1 potency and enhanced selectivity profile across kinase and non-kinase screening panels (Ndubaku et al., 2015) (Figure 5B).

Unfortunately, the addition of lipophilic groups to small molecules is a common tactic that is frequently employed by inexperienced medicinal chemists who are seduced by increased affinity for their biological target of interest, but unaccustomed to the likely risks of aggregation and biological promiscuity, not to mention metabolic instability. Thus, such molecules continue to be synthesized and used and the biology community should be wary of chemical tools with high calculated or measured lipophilicity. This property is captured by LogP values that reflect the relative partitioning between water and octanol and these can easily be provided by chemistry colleagues. Lipophilic compounds can, and should, be readily tested for self-aggregation (McGovern et al., 2003: Irwin et al., 2015) and off-target promiscuity (Eurofins/CEREP, 2017) at a fraction of the cost of wasted efforts on complex *in vivo* and cell-based studies.

#### Pharmacophore Crossing

While protein sequence homology, biological target class membership and/or shared endogenous ligands provide a shortlist of likely suspects for off-target activity, small molecules can still lead an active “night-life” and interact in unanticipated ways with distant parts of the proteome. The increasing power and adoption of ABPP is revealing the potential for off-target pharmacology with current drugs and chemical tools. For example, a recent report highlights a chemical proteomics approach that revealed the enzyme ferrochelatase (FECH) as a surprisingly common off-target of kinase inhibitors; notably, 29 of the 226 clinical kinase inhibitors tested, including approved drugs vemurafenib and neratinib, bind to the protoporphyrin pocket of FECH at low- or sub-micromolar concentrations (Klaeger et al., 2016); this is known as “pharmacophore crossing” (see Box 1).

Several recent studies highlight the discovery of notable, and at first glance surprising, pharmacophore crossing and off-target pharmacology involving unrelated gene families, for both chemical tools and clinical agents that may otherwise have been regarded as selective. In an interesting and topical example, several kinase inhibitors in clinical use have subsequently been demonstrated to exhibit high affinity for bromodomain histone acetyl lysine readers, such that these compounds should now be reclassified as dual kinase-bromodomain modulators at therapeutically relevant exposures (Ciceri et al., 2014, Ember et al., 2014), and appropriate care should be taken in interpreting results with such agents. Notably BI2536, currently marketed as a selective PLK1 inhibitor, demonstrates comparable BRD4 bromodomain affinity (IC_50_ = 25 nM) to the chemical tool JQ1 (IC_50_ = 35 nM); and compound TG10129, currently sold as a selective JAK2 inhibitor (IC_50_ = 6 nM), demonstrates an affinity for BRD4 (IC_50_ = 130 nM) as well as for the kinases FLT3 (IC_50_ = 25 nM) and RET (IC_50_ = 17 nM) sufficient to cast doubt on the pharmacology underlying some of the cellular phenotypes observed when it is used at higher concentrations (Figure 5C) (Ember et al., 2014). Interestingly, a comparative structural analysis of binding modes for multiple compounds exhibiting bromodomain to kinase pharmacophore crossing revealed that diverse molecular interactions may elicit bromodomain affinity, not all of which overlap with interactions required for kinase ATP-binding-site affinity, highlighting a significant medicinal chemistry challenge for chemical probe and drug design.

In another notable clinically relevant example, the Aurora A kinase inhibitor MLN8054 elicited reversible drowsiness as a dose-limiting toxicity in Phase I dose-escalation studies, consistent with its demonstrated affinity for the off-target GABA_A_ α-1 benzodiazepine binding site. Interestingly, MLN8054 contains a benzodiazepine core scaffold; however, a small structural change in the periphery of the molecule abrogated GABA_A_ α-1 benzodiazepine binding and clinical incidence of somnolence in the follow-on candidate alisertib MLN8237 (Figure 5D), further emphasizing the subtlety of fine-tuning specific ligand-protein interactions (Sells et al., 2015).

### Further Cautionary Tales with Chemical Probes in Cancer

#### PARP Inhibitor Iniparib

**Figure 6:**
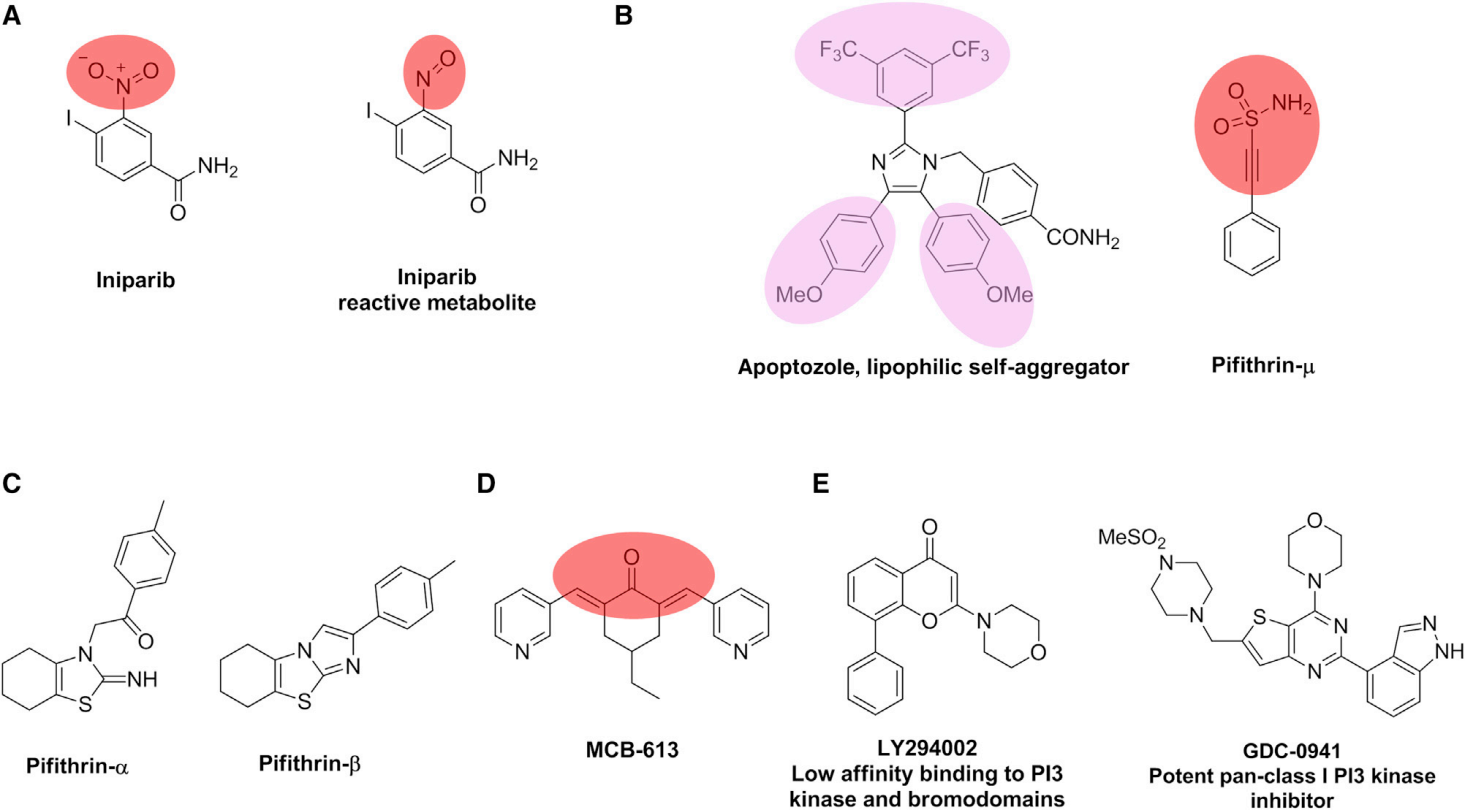
Probes That Elicit a Response in Biochemical or Cell-Based Assays Due to Non-Specific Effects (A) The metabolically unstable and chemically reactive components of iniparib and its reactive metabolite are depicted in red. (B) The substructures contributing to the lipophilicity of apoptazole are highlighted in lilac; the chemically reactive component of pifithrin-μ is highlighted in red. (C) Putative p53 modulator pifithrin-α, which undergoes rapid conversion to pifithrin-β. (D) The chemically reactive components of MCB-613 are highlighted in red. (E) Evolution of the early non-selective pathfinder tool LY294002 to the more potent and selective class I PI3 kinase inhibitor GDC-0941 (pictilisib).

A high profile and cautionary example of an inappropriate and advanced chemical tool is provided by iniparib, a purported poly (ADP-ribose) polymerase (PARP) inhibitor that failed in Phase III clinical trials in breast cancer (Figure 6A) (Sinja, 2014). Iniparib was in the vanguard of PARP inhibitors and was inappropriately progressed through preclinical and clinical studies to test the hypothesis that *BRCA*-mutant tumors would be particularly sensitive to PARP inhibition, shown preclinically with olaparib and other PARP inhibitors considered to be the first exemplification of the concept of synthetic lethality in cancer therapy (Ashworth, 2008). The Phase III clinical trial failure of iniparib raised significant concerns regarding this therapeutic approach; however, subsequent studies, which should have been conducted much earlier, demonstrated that the antitumor activity of iniparib results from its conversion to the highly reactive C-nitroso metabolite (Figure 6A) followed by non-selective modification of cysteine-containing proteins (Patel et al., 2012, Liu et al., 2012). This behavior is due to the presence in iniparib of an aromatic nitro group regarded by many experienced medicinal chemists as a disaster waiting to happen, in both chemical tools and in drug molecules (Blagg, 2006). By notable contrast, validated PARP inhibitors, olaparib and veliparib, have demonstrated remarkable antitumor activity in *BRCA*-mutant models and in corresponding patients, resulting in the recent approval of olaparib for the treatment of women with *BRCA*-mutant ovarian cancer.

#### Molecular Chaperone HSP70

Protein targets with 3D structures that lack well-defined cavities or grooves able to readily accommodate drug-like small molecules (including hard-to-drug cancer proteins such as RAS, MYC, mutant p53, β-catenin, and the molecular chaperone HSP70) are particularly prone to a prevalence of poor-quality chemical tools. The application of biochemical screening techniques will generally identify small molecules that elicit an assay response; however, in many cases, the most likely cause is entirely unrelated to binding at the desired protein cavity or groove, and commonly derives from interference with the assay readout. Furthermore, factors such as compound chemical reactivity, non-specific lipophilic interactions, or aggregation can lead to false-positive readouts as described earlier (Figure 4).

The aforementioned caveat emptor – let the buyer beware – applies equally well here. Furthermore, if the protein under study has been demonstrated to have a clear oncogenic role using biological reagents and techniques, then this scenario is even more worrisome as poor chemical tools generate false excitement and beget further biomedical interest, publications, and grant funding, despite their likely lack of specificity. Numerous reports using biological reagents and techniques built confidence in validation of the molecular chaperone heat shock protein 70 kDa (HSP70) family as a drug target in multiple cancer types and in drug resistance (Powers et al., 2010). This has spawned multiple approaches to discover small-molecule modulators of HSP70 function, many of which should be regarded with suspicion despite the presence of a potentially druggable, although challenging, ATP-binding site in HSP70 (Jones et al., 2016). Notably, apoptozole (Figure 6B) has been reported to possess strong affinity for the HSP70 protein family (Williams et al., 2008); however, investigations using both biochemical and biophysical techniques failed to demonstrate binding of apoptazole to HSP70. Unsurprisingly, consistent with the lipophilic structure of apoptozole (with a preference for a lipid over an aqueous environment of >10 million fold), this agent was found to self-aggregate in aqueous media, and the lipid micelles formed were shown to interact with HSP70 proteins in a non-specific manner (Evans et al., 2015).

A second so-called chemical tool pifithrin-μ (PFT-μ), which is marketed by many commercial vendors as an HSP70 modulator, is, we propose, equally suspect. The chemical structure of PFT-μ contains a potentially reactive scaffold: a carbon-carbon triple bond (acetylene) directly attached to an electron-withdrawing group (sulfonamide) (Figure 6B) (Blagg, 2006). This motif clearly signposts to chemists its potential to covalently modify proteins through reaction with cysteine thiols. Indeed, several other reports in reputable journals highlight potentially interesting pharmacology for PFT-μ, which may further hint at non-specific activity: an interaction with p53 that inhibits the binding of p53 to mitochondria (Strom et al., 2006) and PFT-μ-driven p53-independent apoptosis in B-chronic lymphocytic leukemia cells via the mitochondrial pathway (Steele et al., 2009). Given the potential for non-specific covalent modification of proteins by PFT-μ we recommend caution in its use. The very similarly named pifithrin-α (PFT-α) has also been reported to inhibit p53 function (Komarov et al., 1999). PFT-α has a chemical structure entirely different from PFT-μ; however, it is an equally suspect chemical tool which undergoes rapid conversion to pifithrin-β (PFT-β) (Figure 6C). The use of both PFT-α and PFT-β has been discredited for over 10 years (Walton et al., 2005); however, a Google Scholar search for PFT-α and p53 revealed 336 references published in 2016.

#### KRAS and Autophagy

Considerable efforts have been made to tackle the cancer driver KRAS, a bona fide oncoprotein that lacks clearly druggable cavities or grooves, and a number of early studies concluded that *KRAS*-mutant tumors become addicted to autophagy. However, a combined group from Novartis and Pfizer recently demonstrated that RNAi-mediated knockdown of genes critical to autophagic function across 47 *KRAS*-mutant and wild-type cell lines had no effect on cell growth irrespective of *KRAS* status (Eng et al., 2016). Similarly, CRISPR knockout of genes involved in autophagy, both *in vitro*, in cell lines and in animal models, had no effect. Furthermore, chloroquine, a lysosomotropic agent commonly used as a claimed chemical probe to inhibit autophagy function, and also a more potent analog thereof, were shown to elicit antiproliferative effects in *KRAS*-mutant cancer cell lines in the absence of a functioning autophagy pathway, thereby devalidating the hypothesis that *KRAS*-mutant tumors are addicted to autophagy and devalidating chloroquine as a chemical probe for interrogating autophagy function (Eng et al., 2016). This example further illustrates the need for the integrated use of biological and chemical tools to test mechanistic hypotheses: the deficiencies of either approach can be highlighted by the other.

#### Pan-Steroid Receptor Co-activator MCB-613

In some cases, particularly startling exemplars trigger lively scientific debate among the medicinal chemistry and chemical biology community. For example, MCB-613, a pan-steroid receptor co-activator (SRC) was reported to overstimulate cancer cells leading to cell stress and death (Wang et al., 2015). But use of MCB-613, and the related bis-chalcone class of compounds, received criticism in the topical “In The Pipeline” blog (Lowe, 2016). Like PFT-μ, MCB-613 is chemically reactive and derives from a series of molecules (bis-chalcones) that have a long history of broad cell-based biological activity, most likely due to non-specific covalent adduct formation with a plethora of cellular proteins (Figure 6D). Critically important is the realization that the use of molecules, such as PFT-α, PFT-μ, or MCB-613, to test specific biological hypotheses is fraught with danger owing to the likelihood of confounding off-target biological activity. Thus, the discovery of such molecules as hits in numerous biological screens is entirely unsurprising for the same reason, and should trigger alarm bells to all concerned, including journal referees and grant-funding bodies.

#### Probe Evolution

There are multiple examples where chemical probes have evolved from what are initially fit-for-purpose, but not optimal, “pathfinder” molecules to what eventually are high-quality chemical probes suitable for use in cell-based and animal studies (Workman and Collins, 2010). Despite this, biologists commonly continue to use the earlier poorer-quality probes which gather even more citations in a non-virtuous cycle. The evolution of chemical probes used to study the roles of PI3 kinase is a useful exemplar. LY294002 (Figure 6E) was originally described in 1994 as a PI3 kinase inhibitor, and its use has been reported in 30,000 scientific publications. At the time of its initial disclosure LY294002 represented the best available tool with which to study the kinase function of PI3 kinase and it proved to be an archetypal early pathfinder probe (Workman and Collins, 2010). However, more recent research has revealed major limitations of LY294002, most notably its weak affinity for PI3 kinase (Ki = 1.6 μM) and its promiscuous off-target pharmacology beyond the kinome at concentrations required to elicit PI3 kinase inhibition, including effects on the bromodomain containing proteins BRD2, 3 and 4 (Dittmann et al., 2014). With the discovery of more potent and selective PI3 kinase inhibitors, such as pictilisib (GDC-0941), a pan-class I PI3K inhibitor; GDC-0980, a dual PI3K/mTOR inhibitor; and idelalisib (GS-1101 or CAL-101), a PI3Kδ inhibitor (Yap et al., 2015), biologists should no longer be using LY294002. However, entering PI3 kinase and LY294002 into Google Scholar generated 1,190 results for the year 2016 alone, and commercial vendors continue to sell LY294002 as a PI3 kinase inhibitor.

The pathfinder probe ABT-737 was the first small molecule to target the BH3 domain of the B cell lymphoma class of proteins that regulate apoptosis. While ABT-737 had activity across both pro- and anti-apoptotic family members, findings with this compound and its successor ABT-263 (navitoclax) were critical to the subsequent discovery and successful clinical development of ABT-199 (venetoclax), which selectively inhibits the B cell lymphoma 2 (BCL2) family member. This story is now used as a case study of chemical probe evolution on the not-for-profit expert review-based Chemical Probes Portal (www.chemicalprobes.org; see later for more discussion) to illustrate how historic compounds can and should be superseded by improved chemical probes (Chemical Probes Portal, 2017a, Chemical Probes Portal, 2017b).

### Select Your Chemical Probe Carefully

With the above considerations in mind, we recommend that, in order to gain help and advice in selecting chemical tools appropriate for testing a biological hypothesis, and to avoid the pitfalls described herein, biologists should consult with experienced and expert medicinal chemists and/or chemical biologists familiar with issues around chemical and pharmacological properties. Many authors have articulated the ideal attributes of chemical tools and, to help biologists, we provide a checklist of considerations (Box 3) and also guidance on dos and dont's (Figure 2). All attributes may not be immediately achievable and an evolution of probe quality or “fitness” is to be expected, particularly for unexplored protein classes where early pathfinder probes are valuable (Frye, 2010, Workman and Collins, 2010). In such cases, biological results should be interpreted accordingly and the concomitant use of appropriate biological tools is recommended to build confidence and consensus in the robustness of experimental outcomes and the interpretation thereof. Furthermore, as the quality of chemical probes against a particular biological target evolves, it is important that the biology community progressively adopts the new improved probes for their studies to maximize the interpretability and value of the generated data.

### Recommendations for Probe Selection: 2017 and Beyond

Use of suboptimal, or frankly poor, chemical probes is polluting the scientific literature and wasting time and resources. Such bad practice is contributing to concerns about the reproducibility and robustness of scientific findings and the validation of biological targets (Collins and Tabak, 2014, Frye et al., 2015). Loose standards in the design and use of chemical probes are leading to potentially serious errors in biomedical research studies.

Over the last decade, both academic and industrial research groups have dramatically increased their efforts to produce chemical probes acting on a wide range of target proteins. Many probes emerging from these efforts have fulfilled expectations, acting as powerful research tools to understand biology and providing seeds to spur the development of new medicines. But as the use of chemical probes has increased, it has become clear that many such tools have significant limitations, and are often compromised by fatal flaws.

It is quite common to see researchers continuing to use out-of-date probes to investigate a target protein, when higher-quality chemical probes already exist. In many instances, chemical probes may affect proteins other than those claimed, often exhibiting a few critical or multiple off-target effects. With significant advances in chemoproteomics techniques, such as ABPP (Willems et al., 2014), and greater coverage of the druggable genome in broad screening panels, more examples are emerging of off-target activity in protein classes entirely different from the intended class, and this will likely increase as use of broader profiling expands. As we have seen, there are examples of drugs progressing to the clinic that are inactive against the claimed biological target. In extreme cases, unfortunately not at all uncommon, chemicals that are claimed as useful probes may be indiscriminate in their actions, affecting a very large number of proteins in the cell and rendering them essentially useless as tools for biomedical research. Urgent corrective action is needed.

Although the volume of the call to improve selection and use of chemical probes has been rising within the expert chemical biology and medicinal chemistry communities, and through publications in those fields (Frye, 2010, Workman and Collins, 2010, Bunnage et al., 2013, Blagg and Workman, 2014, Garbaccio and Parnee, 2016), our particular purpose here is to reach out beyond this expert group and increase awareness across the user community of biologists, including cancer researchers, who may unwittingly be promulgating bad practice with chemical probes.

On a more optimistic note, the chemical probe community recognizes the issues and is working toward better standards. The biology community can help by consciously and critically selecting the best tools for the job. This is important, not only to ensure that the data generated within a biology team are relevant to the hypothesis under test but also to make certain that publications resulting from such studies lay solid foundations upon which others can build: a fundamental tenet of scientific progress (Kaelin, 2017).

Key points in this article, captured as a checklist in Box 3 and Figure 2 (see also Blagg and Workman, 2014, Arrowsmith et al., 2015), include the importance of experimental evidence for potency and selectivity. This requires evidence not only from cell-free biochemical assays, such as broad profiling against potential off-targets, but also critical assessment in multiple relevant cellular assays and, if appropriate, whole animal models. Also, to further minimize the risk of reaching false conclusions from off-target effects, the use of carefully designed controls involving at least two different chemical probes with distinct chemical structures, as well as inactive analogs, is strongly recommended. Orthogonal biological and genetic approaches and controls are also important (Figure 2).

Chemical probes need to dissolve well and be stable in water, penetrate into cells, show clear evidence of target engagement and relevant biochemical pathway modulation in cells, and be readily available to researchers in pure form, for example from specialist commercial vendors who can provide evidence of identity and purity. Additional characteristics are needed if the chemical is to be used for research in animals, which requires features that are closer to those of a drug, such as distribution to tissues and reasonable half-life.

Indeed, it is increasingly clear that in some respects the criteria for high-quality chemical probes often need to be more stringent than for a drug to be used in patients, especially in regard to selectivity. This is because it is necessary for some drugs to act on several targets (polypharmacology) in order to deliver the desired clinical benefit, whereas chemical probes need to be much more selective to ask specific biological questions. It is often not appreciated by biologists that a considerable amount of work is required to achieve a truly high-quality probe; chemical compounds identified by HTS are usually just the starting point and if used “as is” can be the source of many of the problems discussed here.

A noteworthy recent development has been the establishment of the Chemical Probes Portal (Chemical Probes Portal, 2017c), which provides the research community with expert guidance in the selection and proper usage of chemical probes for specific protein targets with inclusion of recommended probes and crowdsourced comments, as well as information on historically relevant compounds. This portal now includes over 400 probes and rising, each of which is subject to expert peer review comment and is accompanied by an inventory of relevant properties and guidance on their appropriate use. In addition, the Chemistry in Cancer Research (CICR) community of the AACR is sponsoring education sessions, exemplifying good practice in the use of chemical probes, including at future AACR annual meetings (American Association for Cancer Research, 2017).

We recommend the use of the Chemical Probes Portal, and the checklist in Box 3 and Figure 2, so that researchers can ask key questions about the quality of chemical probes. Both should also be useful to journal editors and to peer reviewers of publications and research grants. When new chemical probes are submitted for publication, selection of appropriate reviewers who are experienced in the discovery and application of such tools is essential. We urge commercial vendors to provide accurate and up-to-date information on the properties of probes, such as their selectivity and other fitness factors, and to make available matched inactive control compounds.

We need to maximize the promise and minimize the peril of chemical probes (Arrowsmith et al., 2015), and this requires the broad research community to use high-quality chemical probes that have been critiqued with equivalent rigor to biological reagents. It is time to put our house in order – and biologists as well as chemists have an important responsibility to do so.
