# Choose and Use Your Chemical Probe Wisely to Explore Cancer Biology

**DOI:** 10.1016/j.ccell.2017.07.010

**Published:** 2017-08-14

**Authors:** Julian Blagg, Paul Workman

(Cancer Cell *32*, 9–25; July 10, 2017)

In the originally published version of this article, the authors mistakenly depicted an incorrect chemical structure for BI2536 in Figure 5C. The original version had the position of an ethyl substituent and a carbonyl group switched. The figure has now been updated with the correct chemical structure and is shown here and in the article online. This transcriptional error does not affect the conclusions in the Perspective. The authors apologize for this error and any confusion that it may have caused.

**Figure 5:**
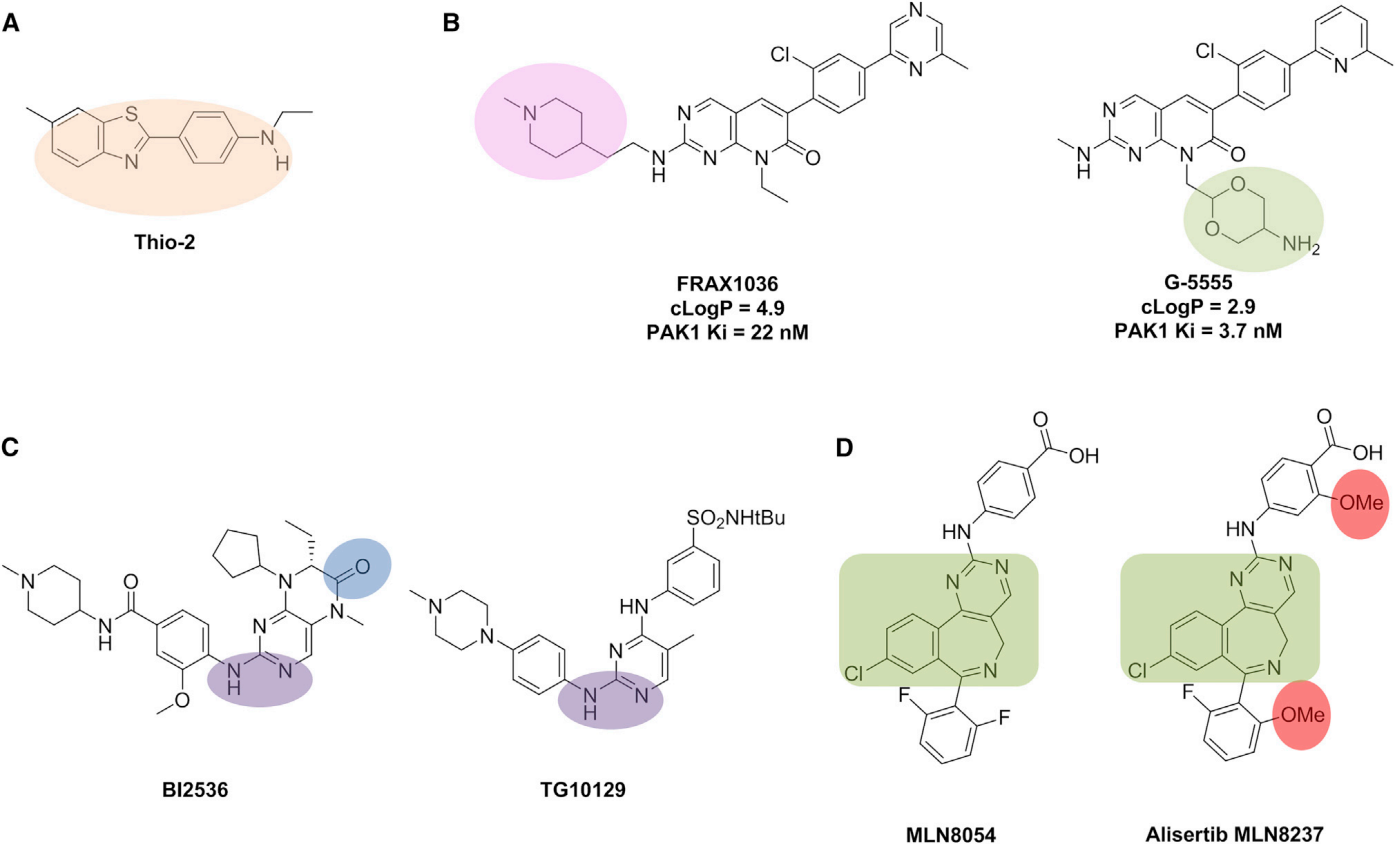
Thio-2, Lowering Lipophilicity and Pharmacophore Crossing (corrected)

**Figure 5:**
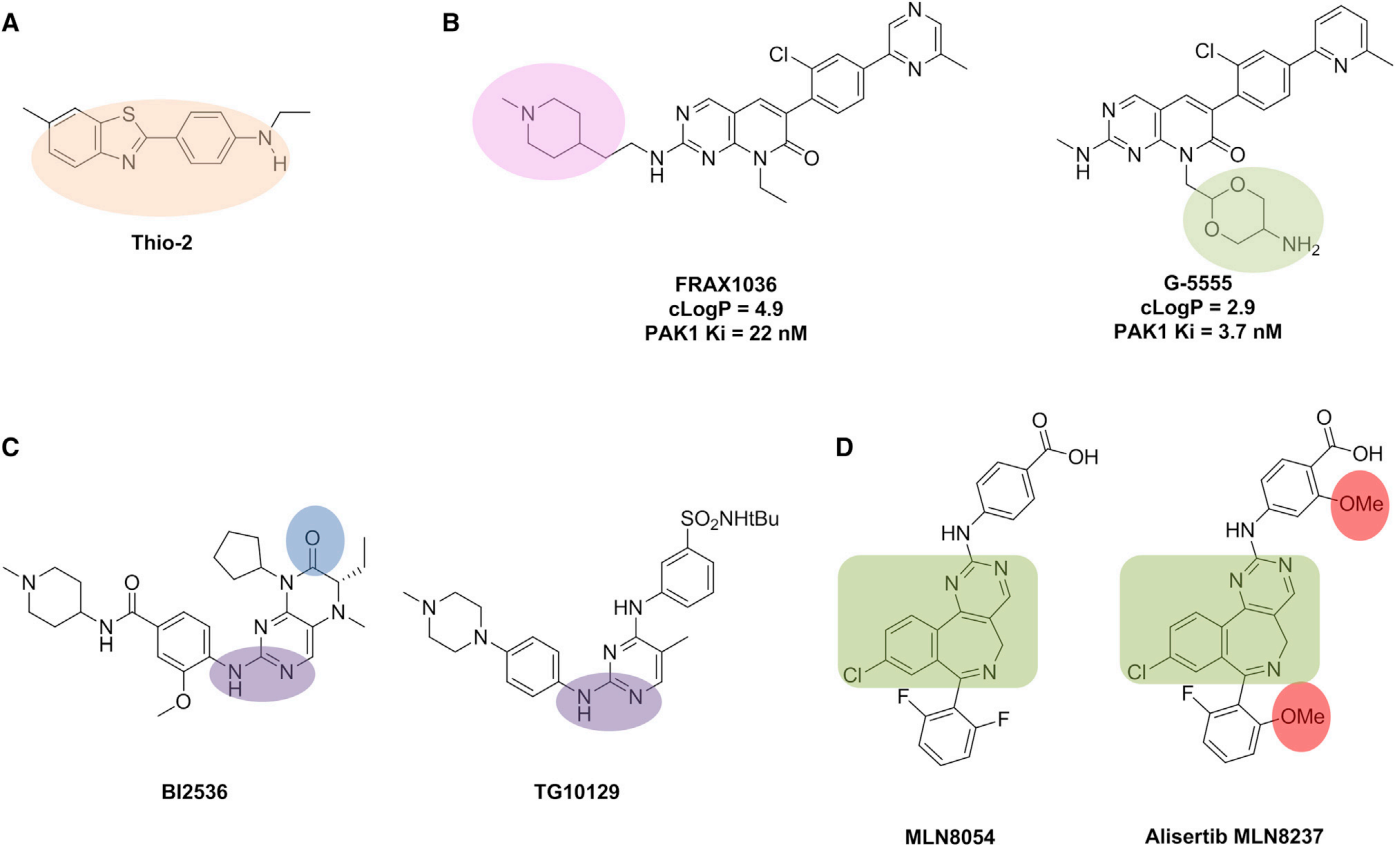
Thio-2, Lowering Lipophilicity and Pharmacophore Crossing (original)

